# Identification of Novel Mutations in *Spatacsin* and *Apolipoprotein B* Genes in a Patient with Spastic Paraplegia and Hypobetalipoproteinemia

**DOI:** 10.1155/2015/219691

**Published:** 2015-05-07

**Authors:** Leema Reddy Peddareddygari, Raji P. Grewal

**Affiliations:** ^1^The Neuro-Genetics Institute, 501 Elmwood Avenue, Sharon Hill, PA 19079, USA; ^2^Neuroscience Institute, Saint Francis Medical Center, 601 Hamilton Avenue, Trenton, NJ 08629, USA

## Abstract

Complicated hereditary spastic paraplegia (HSP) presents with complex neurological and nonneurological manifestations. We report a patient with autosomal recessive (AR) HSP in whom laboratory investigations revealed hypobetalipoproteinemia raising the possibility of a shared pathophysiology of these clinical features. A lipid profile of his parents disclosed a normal maternal lipid profile. However, the paternal lipid profile was similar to that of the patient suggesting autosomal dominant transmission of this trait. Whole exome sequence analysis was performed and novel mutations were detected in both the *SPG11* and the *APOB* genes. Genetic testing of the parents showed that both *APOB* variants were inherited from the father while the *SPG11* variants were inherited one from each parent. Our results indicate that, in this patient, the hypobetalipoproteinemia and spastic paraplegia are unrelated resulting from mutations in two independent genes. This clinical study provides support for the use of whole exome sequencing as a diagnostic tool for identification of mutations in conditions with complex presentations.

## 1. Introduction

Hereditary spastic paraplegias (HSPs) are a genetically heterogeneous group of neurodegenerative disorders with autosomal dominant, recessive, or an X-linked pattern of inheritance. Clinically they can be classified as pure (or uncomplicated) form or the complicated form of HSP where spasticity may be associated with a combination of neurological or nonneurological manifestations. These can include cerebellar ataxia, dysarthria, mental retardation, optic atrophy, retinitis pigmentosa, hearing loss, a thin corpus callosum, or peripheral neuropathy. HSP can also be genetically classified depending on their specific locus which at present ranges from SPG1 to SPG72 (http://neuromuscular.wustl.edu/spinal/fsp.html).

Familial hypobetalipoproteinemia (FHBL) is an autosomal dominant condition characterized by low plasma concentrations of total cholesterol, low-density lipoprotein cholesterol, and apolipoprotein B (apoB). Mutations in several different genes can cause hypobetalipoproteinemia, the most common of which are a result of truncating mutations in the* APOB* gene [1].

Several different mechanisms have been proposed regarding the pathogenesis of HSP including defective subcellular transportation, mitochondrial malfunction, and increased oxidative stress. Most recently the identification of 1088C > T (S363F) mutation in exon 5 of* cytochrome P450-7B1* in SPG5 disease locus provides a link between cholesterol metabolism and neuronal degeneration in HSPs [2]. The association of HSP with familial hypobetalipoproteinemia is rare and has been reported only once previously [3]. Although a disease causing mutation in this family was identified in the* APOB* gene, they were not able to identify the genetic cause of HSP.

We report a patient with HSP and associated hypobetalipoproteinemia in whom whole exome sequencing was performed to identify the disease causative mutation(s) after initial diagnostic testing for HSP was negative.

## 2. Case Presentation

This is a 30-year-old man who was well until the age of 13 years when he started tripping and falling. He was observed to be dragging his feet and developed a clumsy gait. These symptoms progressed and he started to use a cane at the age of 16, a walker at the age of 18, and, ultimately, a wheelchair at the age of 24 years. During this time period he had numerous neurological evaluations and was diagnosed with spastic paraparesis. He has had no associated complaints of pain, numbness, bowel or bladder symptoms, seizures, visual abnormalities, or vertigo. In the last few years, he has developed symptoms of dysarthric speech and clumsiness of his upper extremities. He was diagnosed with attention deficit disorder and further evaluations showed IQ scores (Wechsler Intelligence Scale for Children) of 107 (verbal), 100 (performance), with an overall score of 103. He went on to complete high school and postsecondary education.

He is the product of a normal full term pregnancy and although both parents are of Italian origin, there is no consanguinity. There is no family history of any neurological disorder on either the paternal or maternal branches of the family. The patient has no siblings.

General physical examination revealed no abnormalities. Neurological examination performed showed a minimental status score of 30/30. His speech was dysarthric but otherwise the cranial nerve and sensory examinations were normal. The stretch reflexes were diffusely pathologically brisk and he had sustained clonus at the ankles with bilateral extensor responses. Motor examination revealed a marked hypertonia across all joints bilaterally. Both lower extremities were extended at the knee joint and very spastic. Power was full in the upper extremities (MRC Grade 5/5) and reduced in the lower extremities (MRC Grade 3/5 distally). He could not stand unassisted. A neurological examination was performed on both parents and revealed no abnormalities.

During the course of his medical care many investigations were performed and the following tests were either normal or negative: routine serum chemistries, cell count and differential, creatine phosphokinase, renal and thyroid function studies, and serology for HTLV1.

In addition, magnetic resonance imaging studies of his spinal cord and brain were normal. An electromyography indicated no evidence of neuropathy or myopathy.

A lipid profile of the patient had been performed as part of routine screening and was abnormal. Subsequently, lipid profiles of both parents were performed (Table 1).

## 3. Genetic Testing

All of the genetic investigations performed on this patient and his family members were done after informed consent was obtained following local IRB policies and procedures. Prior genetic testing performed at a number of commercial laboratories was negative for specific genes tested, including* DYT1, SPG3A, SPG4,* and* NIPA1*. However, the* spastic paraplegia 11* (*SPG11*) gene had not been tested. We elected to perform whole exome sequencing through a commercial lab using Illumina HiSeq 2000 platform; sequence capture was done using Agilent SureSelect. The paired-end raw reads were aligned to the hg19 (USCS version) human genome reference and variant list compiled. The raw variant list was analyzed for variants in the known SPG genes and since the patient's lipid profile was consistent with hypobetalipoproteinemia, the variants in* APOB* gene were also analyzed.

**Figure 1:**
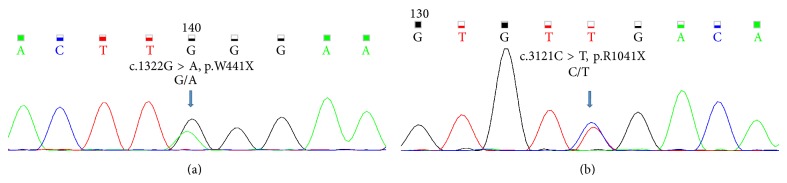
Sangers sequencing results of the* SPG11* (NM_025137.3) variants identified by whole exome sequencing of the patient. (a) Showing the c.1322G > A mutation in exon 6 and (b) showing the c.3121C > T mutation in exon 17 of the* SPG11* gene.

The nucleotide level variant analysis of SPG genes revealed two novel variants on chromosome 15 located in* SPG11* gene. Both of the single nucleotide polymorphisms (SNPs) were reconfirmed in the patient by Sanger sequencing (Figure 1). One variant at genomic position, 44905652, resulted in a heterozygous c.1322G > A change in exon 6 (ENSE00001183238, NM_025137). This change results in premature truncation of the spatacsin protein p.W441X (Figure 1(a)). The second variant at genomic position 44943823 results in a c.3121C > T change in the exon 17 (ENSE00001287244, NM_025137) of the* SPG11* gene also resulting in premature truncation of the spatacsin protein p.R1041X (Figure 1(b)).

**Figure 2:**
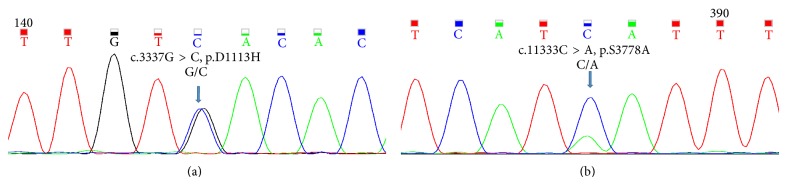
Sangers sequencing results of the* APOB* (NM_000384.2) variants identified by whole exome sequencing of the patient. (a) Showing the variant c.3337G > C in exon 22 and (b) showing the variant c.11333C > A in exon 26 of the* APOB* gene.

The nucleotide level variant analysis of* APOB gene* on chromosome 2 revealed two SNPs that were reconfirmed by Sanger sequencing (Figure 2). A previously described change at genomic position 21238413 that causes c.3337G > C change in exon 22 (ENSE00000542198, NM_000384), resulting in a missense mutation from substitution of amino acid, p.D1113H (Figure 2(a)). A novel SNP was also identified, a G to T change at genomic position 21228407. This heterozygous c.11333C > A variant in the exon 26 (ENSE00001183453, NM_000384) results in a stop codon leading to premature truncation of the protein p.S3778X (Figure 2(b)).

**Figure 3:**
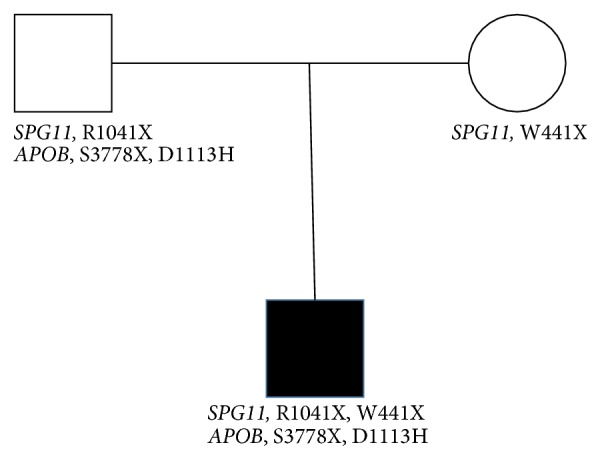
Pedigree of the family showing the parents and the affected individual with mutations in the* SPG11* and* APOB* gene. The son is compound heterozygous for* SPG11* mutations, inheriting one variant from each of the parents. Both* APOB* variants were inherited from the father.

The patient is a compound heterozygous for two truncating mutations in the* SPG11* gene. Sanger sequencing of the exons containing these individual SNPs on DNA obtained from his parents indicates that the patient has inherited the p.W441X mutation from the mother and p.R1041X mutation from the father (Figure 3). The parents were also tested for the* APOB* mutations and no mutations were detected in the mother. However, analysis of the paternal DNA shows the presence of both* APOB* variants (Figure 3).

## 4. Discussion

Spastic paraplegia 11 (SPG11) is an autosomal recessive disorder caused by loss of function mutations in the* spatacsin* (*SPG11*) gene on chromosome 15q21.1. This patient's phenotype is consistent with that described in previously reported patients with genetically confirmed SPG11 (http://neuromuscular.wustl.edu/spinal/fsp.html#spgmu). There are 98 pathogenic/likely pathogenic SNPs that have been reported (http://www.ncbi.nlm.nih.gov/clinvar/). Our patient inherited two losses of function mutations, one from each parent. Both of these mutations are novel and result in premature truncation of the protein strongly suggesting that they are disease producing mutations causing HSP.

The* APOB* gene is translated into both apoB-100 and apoB-48 proteins. The shorter apoB-48 protein is produced after RNA editing of the apoB-100 transcript at residue 2180 (CAA→UAA), resulting in a stop codon producing early termination. The majority of reported* APOB* mutations result in premature truncation of the protein. In heterozygous* APOB* mutation, the normal apoB-100 product is produced at a lower rate while truncated apoB is cleared too rapidly resulting in the low apoB-100 levels [4]. Nonsynonymous, nontruncating* APOB* mutations have also been reported [5, 6]. It was reported that a nontruncated mutant protein can result in impaired secretion compared with the normal secretion of the mutant truncated proteins [7]. All of the previously reported nontruncating mutations are observed in residues 292–593.

Heterozygotes for FHBL have less than half-normal LDL-cholesterol and apoB concentrations, whereas homozygotes have extremely low or undetectable LDL-cholesterol and apoB levels. Both the father and the son have variants in exons 22 and 26. The exon 22 variant causes a substitution of aspartic acid at position 1113 with histidine and affects both apoB-100 and apoB-48. This SNP has been reported in the 1000-genome project with a minor allele frequency of G = 0.003/7 and this change is predicted to be damaging to the protein by both Sift (score 0.02) and PolyPhen (score 0.999) analysis (http://www.ensembl.org/index.html, rs12713844). This variant does not involve the N-terminal *βα*1 domain (residues 1–930) of the protein which is important for effective secretion and in subpopulation of Utah residents (CEPH) with Northern and Western European ancestry the frequency of allele G is reported to be as high as 2%. Therefore APOB p.D1113H may not be a disease producing variant. The exon 26 (ENSE00001183453, NM_000384) encodes amino acids 1406 to 3931. The exon 26 variant, 3778 (TCA→TAA), inherited by the patient from his father results in a stop codon leading to premature truncation of the protein p.S3778X. Therefore, it is more likely that the disease causing mutation in the father and son is this exon 26 variant which does not affect apoB-48 but does cause premature truncation of the apoB-100 at residue 3778. In previous reports of heterozygotes for point mutations located in the exon 26 of the* APOB gene* it was found that the clinical manifestations of FHBL are dependent on the size of the resultant truncated apoB [7]. This variant likely accounts for the low LDL-cholesterol, total cholesterol, and apoB-100 observed in the father and son.

When the patient was first encountered, similar to the previously reported family [3], it was hypothesized that there may be connection between the abnormal cholesterol profile and spasticity. However, the genetic analysis performed indicates that these are disparate clinical features.

This case provides support for the value of whole exome sequencing as a diagnostic tool for identification of mutations in conditions where the commercial testings were negative. We were able to identify the genetic cause for hereditary spastic paraplegia and hypobetalipoproteinemia in this patient presenting with a rare combination of autosomal recessive progressive spastic paraparesis and autosomal dominant hypobetalipoproteinemia with normal triglycerides.

## Figures and Tables

**Table 1:** Lipid profile values of the patient and his parents.

Lipid profile	Patient	Mother	Father	Normal range
Total cholesterol	82 mg/dL	214 mg/dL	117 mg/dL	<200 mg/dL
Triglycerides	104 mg/dL	163 mg/dL	120 mg/dL	<150 mg/dL
HDL	39 mg/dL	51 mg/dL	50 mg/dL	≤40 mg/dL
VLDL	17 mg/dL	28 mg/dL	17 mg/dL	<30 mg/dL
LDL	25 mg/dL	135 mg/dL	50 mg/dL	<130 mg/dL
apoB-100-calc	41 mg/dL	109 mg/dL	53 mg/dL	<109 mg/dL
